# Values of Importance to Patients With Cardiovascular Disease as a Foundation for eHealth Design and Evaluation: Mixed Methods Study

**DOI:** 10.2196/33252

**Published:** 2021-10-22

**Authors:** Britt E Bente, Jobke Wentzel, Rik GH Groeneveld, Renée VH IJzerman, David R de Buisonjé, Linda D Breeman, Veronica R Janssen, Roderik Kraaijenhagen, Marcel E Pieterse, Andrea WM Evers, Julia EWC van Gemert-Pijnen

**Affiliations:** 1 Department of Psychology, Health and Technology Centre for eHealth and Wellbeing Research University of Twente Enschede Netherlands; 2 Research Group IT Innovations in Health Care Windesheim University of Applied Sciences Zwolle Netherlands; 3 Unit of Health, Medical, and Neuropsychology Faculty of Social and Behavioural Sciences Leiden University Leiden Netherlands; 4 Department of Cardiology Amsterdam University Medical Center Academic Medical Center Amsterdam Netherlands; 5 Department of Cardiology Leiden University Medical Center Leiden Netherlands; 6 Vital10 Amsterdam Netherlands; 7 NDDO Institute for Prevention and Early Diagnostics (NIPED) Amsterdam Netherlands; 8 Department of Psychiatry Leiden University Medical Center Leiden Netherlands

**Keywords:** patient values, health behavior, lifestyle, mobile app, user-centered design, eHealth, cardiovascular disease, behavior, app, design, cardiovascular, evaluation, platform, support, intervention

## Abstract

**Background:**

eHealth interventions are developed to support and facilitate patients with lifestyle changes and self-care tasks after being diagnosed with a cardiovascular disease (CVD). Creating long-lasting effects on lifestyle change and health outcomes with eHealth interventions is challenging and requires good understanding of patient values.

**Objective:**

The aim of the study was to identify values of importance to patients with CVD to aid in designing a technological lifestyle platform.

**Methods:**

A mixed method design was applied, combining data from usability testing with an additional online survey study, to validate the outcomes of the usability tests.

**Results:**

A total of 11 relevant patient values were identified, including the need for security, support, not wanting to feel anxious, tailoring of treatment, and personalized, accessible care. The validation survey shows that all values but one (value 9: To have extrinsic motivation to accomplish goals or activities [related to health/lifestyle]) were regarded as important/very important. A rating of very unimportant or unimportant was given by less than 2% of the respondents (value 1: 4/641, 0.6%; value 2: 10/641, 1.6%; value 3: 9/641, 1.4%; value 4: 5/641, 0.8%; value 5: 10/641, 1.6%; value 6: 4/641, 0.6%; value 7: 10/639, 1.6%; value 8: 4/639, 0.6%; value 10: 3/636, 0.5%; value 11: 4/636, 0.6%) to all values except but one (value 9: 56/636, 8.8%).

**Conclusions:**

There is a high consensus among patients regarding the identified values reflecting goals and themes central to their lives, while living with or managing their CVD. The identified values can serve as a foundation for future research to translate and integrate these values into the design of the eHealth technology. This may call for prioritization of values, as not all values can be met equally.

## Introduction

On being diagnosed with a cardiovascular disease (CVD), patients must learn to manage changes in their everyday life due to their disease and its treatment. This new behavior to improve patients’ lifestyle includes following a strict medication regime and learning to self-monitor their health [1]. The aimed health behaviors include, for example, eating a healthy diet; taking regular physical activity; reducing or quitting smoking and alcohol intake; and reducing the body weight, blood pressure, and blood cholesterol [1]. Patients often need support during the period of acquiring new self-management skills and lifelong health maintenance behaviors.

eHealth interventions can support patients in the self-care tasks and the required lifestyle changes. Technology-facilitated health care may offer ubiquitous, ongoing, and personal guidance that supports changes in patient behavior. A recent review showed that eHealth interventions, aimed at CVD self-management, help patients to monitor symptoms, provide information, and provide contact with a care provider in case of questions [2]. In chronic care, one of the challenges is how patients can reach a realistic balance between treatment goals and optimal lifestyle changes, while considering their capability, needs, and wishes [3,4]. To do this, eHealth technology that supports patients with these tasks should be designed via a user- or patient-centered approach. This contributes to better uptake and adherence to lifestyle change, and improve health outcomes [5]. However, achieving long-lasting effects is a challenge [6], and therefore, critical reflection on the development and evaluation of eHealth technology is needed in practice [6]. Patients should be involved in the development of interventions, so that their goals, wishes, and needs lie at the core of development processes. Then the intervention will be appealing to patients and result in the desired uptake and health outcomes.

Consequently, patient (user) values should be considered at the earliest stages of eHealth technology development [1]. Values capture specific needs and desires of patients, such as desired future state, and the motivations and drivers of their behavior [7]. These values should form the basis for the development and evaluation of eHealth technologies in practice, enabling developers to evaluate and attune design choices, for example, persuasive features [8] and personas [9]. Furthermore, patient values (and other stakeholders’ values) should form the basis for a business model that underpins the implementation of eHealth [2].

This study focuses on identifying values of importance for patients with CVD, within the context of the online “BENEFIT” personal health platform (PHP). This online platform aims to support patients with CVD to adopt and maintain a healthy lifestyle [10]. Users can, for example, self-log their data (eg, body weight or blood pressure), view their progress, receive advices based on their health data, and can contact coaches via a chat when they need support or help. We explore how patients with CVD want to be supported to accomplish the many goals they must set. We aim to answer the following research question: *What values are proposed by patients with CVD to achieve* *permanent lifestyle changes, and which should be considered when designing an eHealth platform to promote a healthy lifestyle*? The identified values will provide a relevant foundation for the development and implementation of new eHealth technologies.

## Methods

### Study Design

In this study, a mixed method design was used, combining secondary analyses of usability tests conducted in 2018 (study 1) with an online survey study carried out in 2020 (study 2). The aim of study 1 was to identify relevant patient values, while the aim of study 2 was to validate and bring a hierarchy in weighted values at the population level. This created a foundation to draw conclusions about values of patients with CVD [10]. The qualitative data (study 1) were from 10 interviews conducted in the context of patients’ usability tests with the online “BENEFIT” PHP. Study 2 was a survey distributed to panel members of Harteraad, a Dutch patient association for CVDs.

### Ethical Approval and Consent to Participate

Study 1 was approved by the University’s Ethical Committee (BCE18142). Participants were informed of the voluntary nature of their participation and confidentially was guaranteed. All participants signed an informed consent. Study 2 was approved by the Psychology Research Ethics Committee (2020-06-05-A.W.M. Evers-V1-2474). Informed consent was provided digitally by signing the consent page before starting the online survey.

### Study 1: Secondary Analysis of Usability Tests and Interviews

#### Data Set

The secondary data set included transcripts of usability tests conducted with 10 patients with CVD. These 10 patients were included in an earlier usability study by convenience sampling. The aim of that study was to evaluate the usability of the BENEFIT PHP. These usability sessions (see Multimedia Appendix 1) were guided with a script of assignments based on the BENEFIT PHP’s main functionalities and goals. Interview questions were structured to include 6 main subjects: background information, experiences with their illness, adjustments in life, support, self-management, and drivers in the self-rehabilitation of patients [11].

#### Data Analysis

First, all verbatim transcripts of the data set were revised by one researcher (BB) to identify quotations about what patients with CVD indicated they needed to maintain a healthy lifestyle. All usable quotes were inductively linked to individual codes among the theme “Needs.” A second researcher (JW) checked 10% of the quotations, and disagreements in coding were discussed. Second, these needs were translated to values (BB). We defined values as “Ideals or interests respondents have, the underlying reason why someone wants or needs what they want or need” [7]. While needs reflected a concrete desire, the values are the (underlying) drivers of such needs, and are usually of a more generic nature. By applying this definition, initial codes were grouped on the value level by determining what underlying value could be the driver of these codes. The second researcher (JW) checked whether all codes identified in the first part fitted with the determined values. All resulting values were discussed within a BENEFIT research team meeting consisting of 8 researchers. All participants were informed about the results.

### Study 2: Online Survey Study

#### Sample and Procedure

The survey was created and distributed via Qualtrics software [12] and formed part of a larger survey study in the BENEFIT project (R. V. H. IJzerman et al, MSc, unpublished data, 2020). Respondents received information and an invitation to participate via email from Harteraad, including a link to the survey. Survey data were collected between June 29 and July 14, 2020.

#### Materials

The 11 identified values were framed as closed question statements. Respondents were asked to rate these values on a 7-point Likert scale (ranging from 1=not important at all to 7=very important).

#### Data Analysis

Descriptive statistics were generated for the questionnaire data using SPSS version 26 (IBM) [13]. Friedman nonparametric test for related samples was applied to test for possible differences in importance ratings between the values. After an initial overall test, post hoc pairwise comparisons were performed. To compensate for multiple testing, the level of significance was set to .005.

### Availability of Data and Materials

The transcribed data are not publicly available due to privacy restrictions but are available from the corresponding author on reasonable request.

## Results

### Study 1: Usability Tests

#### Participant Characteristics

This study included data of 10 participants at 4 different health care facilities (eg, rehabilitation centers and hospital cardiac departments) in the Netherlands. Participant age range was between 35 and 79 years, with 8 female participants (Table 1). Most of the participants assessed their own digital skills as medium to good. Inclusion criteria were kept wide regarding CVD diagnosis, to obtain a representative input from all types of patients with CVD.

A total of 11 patient values were determined based on the indicated needs from the data (Textbox 1) of patients with CVD.

**Table 1:** Demographic characteristics of patient sample group 1.

Participant number	Gender	Age (years)	Digital skills (self-assessed by participants)	Diagnosis	Time since diagnosis
1	Female	79	Low	Cardiovascular disease	2 days
2	Female	72	Low	Cardiovascular disease	2 days
3	Female	Did not state	Good	Heart disease	2 years
4	Female	64	Good	Cardiovascular disease	20 years
5	Male	62	Good	Cardiovascular disease	20 years
6	Male	64	Medium	Vascular disease	2.5 years
7	Female	35	Good	Did not state	Entire lifetime
8	Female	71	Medium	Heart disease	Entire lifetime
9	Female	Did not state	Medium	Cardiovascular disease	Did not state
10	Female	36	Good	Heart disease	Entire lifetime

**Overview of perceived needs and assessed values (bold) of patients with cardiovascular disease.**

**1. To have confidence and self-efficacy in the treatment and ability to achieve goals**

- Need to receive health-related feedback from the health care provider
- Desire to receive knowledge of treatment methods

**2. To be seen as a person rather than as a patient**

- Need to not continuously be treated as a patient by social environment

**3. To not feel fear, anxiety, or insecurity about their health**

- Need to have a feeling of health-related safety
- Need to have taken away fears regarding their health status
- Desire to trust their own knowledge

**4. To preserve a sense of autonomy over their life**

- Desire to have some autonomy during their rehabilitation
- Need to stay in charge
- Need to keep their freedom
- Need to have control over own situation
- Need to feel heard by the health care provider

**5. To receive social support**

- Need to have contact with fellow sufferers
- Need to feel support from social circle
- Need to feel acknowledged and understood by their social circle

**6. To have or maintain a healthy lifestyle**

- Desire to prevent new incidents
- Need to stay healthy
- Desire to use less medication by implementing alternatives (such as a healthy lifestyle)
- Need to receive help with physical activity, staying healthy

**7. To have an overview of personal health data**

- Need to have a clear overview of own health data
- Desire to have all health data in one place
- Need to see relations between medical, lifestyle, and mental health status
- Desire to have a (quantified) display of how they feel at that moment

**8. To perceive low thresholds to access health care**

- Need to be treated or helped quickly
- Desire to receive care/treatment at home
- Need to have personal contact with health care providers
- Need for health care professionals to be easily approachable

**9. To be extrinsically motivated to accomplish goals or activities (related to health/lifestyle)**

- Desire to have a new (health) purpose in life
- Need to be motivated
- Need to be guided and supported through the lifestyle change process

**10. To receive reliable information and advice**

- Need to receive reliable information
- Desire to be advised about validated apps, applicable in their own situation
- Desire to receive help by use of interventions/technology
- Need to receive concrete and easily applicable advice to improve their health
- Need to have a guideline in dealing with their illness (also for patients’ environment)

**11. To receive personalized care**

- Need to be the central focus of the therapy
- Need to have own priorities being recognized
- Need to have a personal approach (no one is the average patient as described in protocols)

#### To Have Confidence and Self-Efficacy in the Treatment and Ability to Achieve Goals

Patients valued having confidence in their health care professionals and the treatment they prescribe. Patients also valued that they could follow the treatment plan or were able to achieve their goals. Patients indicated that, for this, they needed sufficient knowledge about the treatment methods they received. Maintaining close contact with their caregiver was also reported as important; patients needed to be updated about their health progress by the involved caregiver, to feel confident about their health status.

When you have breathing problems, that carries a whole other mental load […] it affects your mental wellbeing. […] In those cases, it is pleasant if you don’t have to wait for an appointment with your general practitioner. […] I have a very nice cardiologist here in the Netherlands, I have his email and I can always call him

Participant 7

#### To Be Seen as a Person Rather Than a Patient

Patients valued not constantly feeling that they were a patient with a disease. Patients appreciated that their health complaints were acknowledged by their family and friends, but they also wanted to feel like the person they were before they were diagnosed with chronic heart disease, without bystanders worrying about them.

Until my death I need top medical care, I’m just trying to be ‘friends’ with my health care provider, or at least communicate on an equal level with him […] because in the end, we are both humans

Participant 10

#### To Not Feel Fear, Anxiety, or Insecurity About Their Health

Patients valued not worrying about their physical condition. They liked to be provided with coping strategies or information that helped them feel safe or less anxious. Many patients reported the need for overcoming their fears of having a new incident or worsening condition, and for this they needed reassurance. Patients had lost trust in their body and wanted to be confident about their new capabilities after CVD diagnosis.

Physically, I actually did have confidence, because I’m still young and the rest of my body works properly […] but to go exercise… that was really in my head. I was really afraid to burden myself again.

Participant 7

#### To Preserve a Sense of Autonomy Over Their Life

Patients valued being in control of their life (eg, being able to make their own decisions). Patients indicated their need to oversee their own health and treatment. They wanted to be involved in shared treatment-related decision making.

I like that [participant referring to a situation in which her doctor asked her about her opinion about prescribed medication] kind of interaction, I’m not the type of patient that accepts a mentality like ‘I am the doctor, and you have to do what we tell you’. No, it’s my life. I do come to the doctors for help if I feel like I need them to stay alive, but only in such a way that it feels right for me.

Participant 10

#### To Receive Social Support

Patients valued being heard, supported, and understood by the people that surround them (eg, family and friends) and that they had an empathetic person to talk to. Patients needed to feel acknowledged and understood, as well as supported by their friends and family. However, not only did they need support from family and friends, but also some indicated the need to share experiences with patients with similar experience.

[Researcher: And what about contact with peers? Do you like that?] Yes, if you search on the internet, you’ll find user forums. In there, you can find information about what others did in similar situations or give recommendations for specific problems.”

Participant 8

#### To Have or Maintain a Healthy Lifestyle

Patients valued maintaining or changing their lifestyle in such a way that new incidents were prevented, and they regained their health. They wanted to use less medication, for example, by becoming more physically active or adjusting their eating patterns. However, patients reported that they needed help and guidance for improving their lifestyle.

I’ve always been very active, and I try to maintain that until I’m very old. And I hope that that [points to heart] will cooperate because it’s important to me. It will be great if I can just save myself…

Participant 3

#### To Have an Overview of Personal Health Data

Patients valued having a central source where they could look up all their health data (eg, measured values of physical and mental well-being and health). They needed a place where all collected information was presented and which provided new insights into their condition (eg, because it gives the opportunity to compare data).

I can imagine that there are many people, especially if their medication changes a lot, who at some point lose the overview. I also had a period in which it became difficult to remember what was prescribed. If you can see all that information at a glance, it would be very useful.

Participant 5

#### To Perceive Low Thresholds to Access Health Care

Patients valued receiving help and being treated quickly and easily, at a health care organization or at home. They wanted to be facilitated to manage their own disease and take action. Patients indicated that they sometimes had questions which were, in their opinion, not important enough to make an appointment with their caregiver. They desired easily accessible contact with caregivers and preferred to have access to health care from home.

Well, most of the questions I have are not that urgent that you need an answer right away; these are often practical things. Look, if there is really something wrong, you should contact the general practitioner or the cardiology department quickly. But if you have practical questions, it is easy to ask them this way, then it is not necessary to ask for a consultation. I think that is useful.

Participant 5

#### To Be Extrinsically Motivated to Accomplish Goals or Activities (Related to Health/Lifestyle)

Patients valued being extrinsically motivated or pushed to do or accomplish things, such as following their treatment or performing activities to achieve a healthy lifestyle (eg, via social pressure). They needed a driving force as guidance through the process. They desired to be led through their rehabilitation, to be motivated to achieve their goals.

[Regarding the participation in an online module] Do I get an email that there is a new module available for me? And what if I decline? Could it be that during my next consult, my physician says something like ‘hey you didn’t participate, why not?’ [Yes, that is possible]. Okay, I like that there is some sort of motivational force behind it.”

Participant 10]

#### To Receive Reliable Information and Advice

Patients valued having understandable, relevant information and advice that is scientifically proven and recommended by physicians (ie, evidence-based information). They needed information on which they could rely on and on which they could repeatedly consult (eg, an information flyer about their treatment). There is also much information on the internet, but patients were not sure which information was trustworthy. Patients needed simple, concrete advice about improving their lifestyle, and a guideline for dealing with their disease.

Well, sometimes there is the problem, that when you visit the doctor for an appointment, you get so much information that you do not remember it at all. For that reason, it is also useful to bring someone else in there, but it would be helpful if you can also look up the information here, that’s very easy.

Participant 5

#### To Receive Personalized Care

Patients valued receiving a personal approach in which their opinion and preferences were considered (eg, personalization or tailoring of treatment choices or technical platform features). They needed to be heard by the caregiver and wanted their priorities to be recognized. Patients needed a personal, relevant approach, because there is no “one size fits all” solution for patients. Not all patients react the same on a treatment prescribed in protocols.

My doctor sometimes says, ‘it is your body, it is your life, if you want to try this medication, we will do that, and if you want to quit them, we will quit’. I am in a clinic where doctors and nurses think along with you about everything. That is very pleasant.”

Participant 10

### Study 2: Online Survey

#### Participant Characteristics

The survey sample consisted of Dutch patients with CVD, who were representatives from the panel of Harteraad. In total, the panel included 2600 members [14], of whom 739 responded and 710 completed the survey (response rate of 28.42%). Respondents’ mean age was 67 years, 57.6% (426/739) of them were male, and 43.4% (321/739) attended a form of higher vocational education or university (Table 2).

**Table 2:** Participants’ characteristics in study 2 (N=739).

Characteristics			Value
Age (years), mean (range)			67 (23-90)
**Gender, n (%)**			
	Men	426 (57.6)	
	Women	284 (38.4)	
	Did not state	29 (3.9)	
**Last treated vascular condition, n (%)**			
	Heart disease	285 (38.6)	
	Vascular disease	86 (11.6)	
	Cardiovascular disease	176 (23.8)	
	Other	152 (20.6)	
	Did not state	40 (5.4)	
**Highest completed education, n (%)**			
	Primary education or less	8 (1.1)	
	Lower vocational education	163 (22.1)	
	Intermediate vocational education	151 (20.4)	
	Senior general or preuniversity secondary school	58 (7.8)	
	Higher vocational education	235 (31.8)	
	University education (bachelor’s, master’s, [post]doctoral)	86 (11.6)	
	Other	9 (1.2)	
	Did not state	29 (3.9)	
**Relationship status, n (%)**			
	No partner	158 (21.4)	
	A partner with whom I do not live	31 (4.2)	
	A partner with whom I live	522 (70.6)	
	Did not state	28 (3.8)	

#### Perceived Importance of Values

Respondents were asked to rate the 11 values found in study 1 on how important each value was to them. Some respondents failed to provide a rating for every value; nonresponse ranged between 98 and 103 missing responses per value. On a scale of importance from 1 to 7, the mean scores ranged between 5.13 (value 9) and 6.32 (value 4), as Table 3 shows. For all values, the median was 6 (important), except for value 9. For this value the median was 5 (slightly important). The mode for every value was 6 (important). Table 3 shows the perceived importance ratings per value.

**Figure 1:**
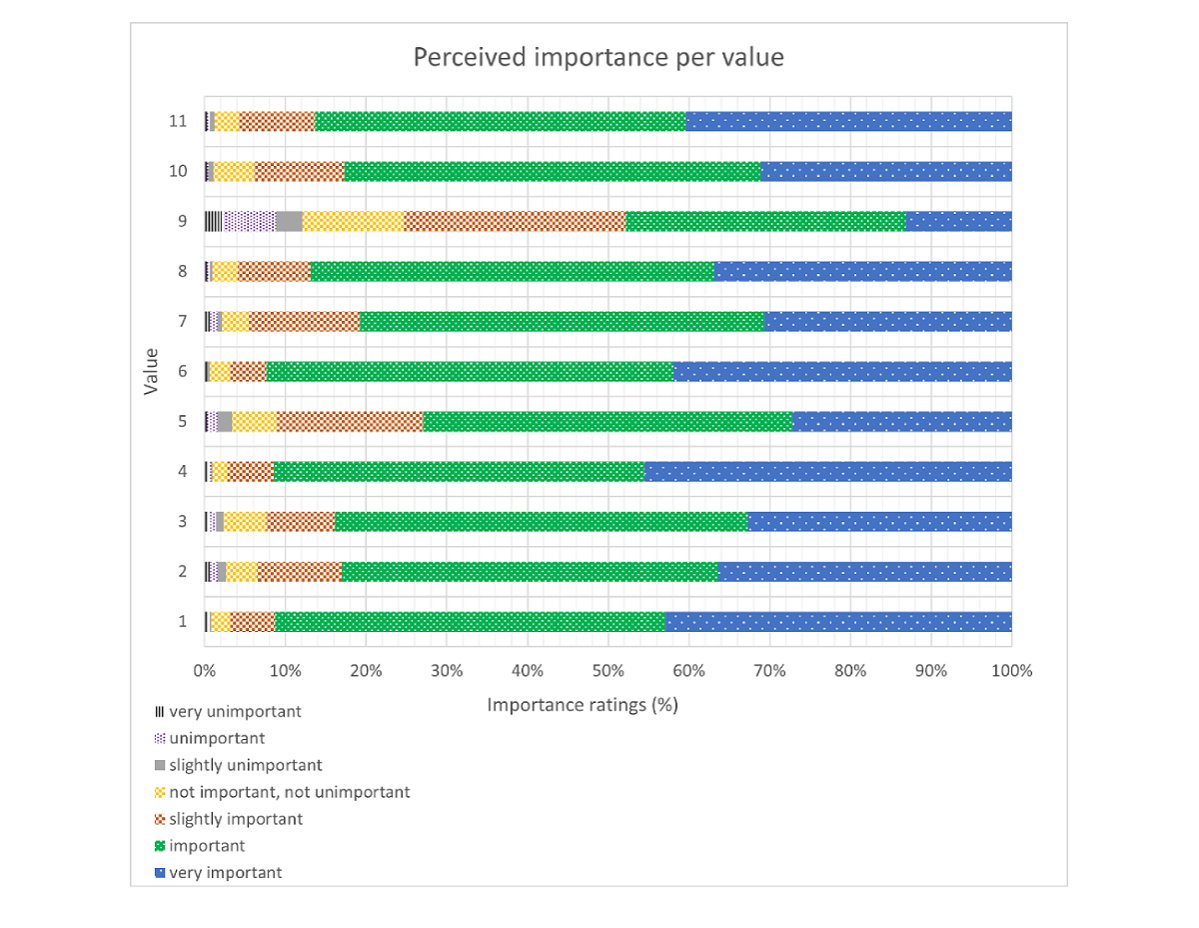
Distribution of perceived importance per value.

The distribution of the rated perceived importance per value is shown in Figure 1. Value 1 (585/641, 91.3%; To have confidence and self-efficacy in their treatment or therapy and their ability to achieve goals), value 4 (586/641, 91.4%; To feel a sense of autonomy of their life), value 6 (590/641, 92.0%; To have or maintain a healthy lifestyle), value 8 (555/639, 86.9%; To have a low threshold to access health care), and value 11 (549/636, 86.3%; To receive personalized care) were considered important or very important by many of the respondents (>85%). For values 1, 4, and 6 this translates to over 90% of the respondents. All other values received ratings of important or very important by most respondents (with ratings for values 2, 3, 5, 7, and 10 ranging between 72.9% and 83.9; ie, value 2: 532/641, 83.0%; value 3: 538/641, 83.9%; value 5: 466/639, 72.9%; value 7: 516/639, 80.8%; value 10: 526/636, 82.7%), except value 9 (To be motivated to accomplish goals or activities [related to health/lifestyle]). This value was rated important or very important by 304/636 (47.8%) respondents. The rating “very unimportant” or “unimportant” was given by less than 2% of the respondents (value 1: 4/641, 0.6%; value 2: 10/641, 1.6%; value 3: 9/641, 1.4%; value 4: 5/641, 0.8%; value 5: 10/641, 1.6%; value 6: 4/641, 0.6%; value 7: 10/639, 1.6%; value 8: 4/639, 0.6%; value 10: 3/636, 0.5%; value 11: 4/636, 0.6%) to all values, except for value 9 (To be extrinsically motivated to accomplish goals or activities [related to health/lifestyle]). This value more often received a rating of “very unimportant” or “unimportant” by 8.8% (56/636) of the respondents.

**Table 3:** Perceived importance ratings per value, ranged from high to low.

Values	Descriptives					
	n	Missing, n (%)	Median^a^	Q1-Q3	Mode^a^	
1. To have confidence and self-efficacy in the treatment and ability to achieve goals	641	98 (15.3)	6 (important)	6-7	6 (important)	
2. To be seen as a person rather than a patient	641	98 (15.3)	6 (important)	6-7	6 (important)	
3. To not feel fear, anxiety, or insecurity about their health	641	98 (15.3)	6 (important)	6-7	6 (important)	
4. To preserve a sense of autonomy over their life	641	98 (15.3)	6 (important)	6-7	6 (important)	
5. To receive social support	639	100 (15.6)	6 (important)	5-7	6 (important)	
6. To have or maintain a healthy lifestyle	639	100 (15.6)	6 (important)	6-7	6 (important)	
7. To have an overview of personal health data	639	100 (15.6)	6 (important)	6-7	6 (important)	
8. To perceive low thresholds to access health care	639	100 (15.6)	6 (important)	6-7	6 (important)	
9. To be extrinsically motivated to accomplish goals or activities (related to health/lifestyle)	636	103 (16.2)	5 (slightly important)	5-6	6 (important)	
10. To receive reliable information and advice	636	103 (16.2)	6 (important)	6-7	6 (important)	
11. To receive personalized care	636	103 (16.2)	6 (important)	6-7	6 (important)	

^a^Likert scales were applied, ranging from 1 (not important at all) to 7 (very important).

As value 9’s ratings resulted in a lower mean, median, and score distribution, a nonparametric test was carried out to investigate if the pattern in ratings differs between the values. The results of Friedman nonparametric test for related samples show that an overall difference in ratings (n=636) was found when comparing all value ratings (*F*_10,625_=856.56, *P*<.001).

## Discussion

### Principal Findings

This study aimed to reveal health-related values of patients with CVD which should be considered when designing an eHealth platform that supports patients to achieve and maintain a healthy lifestyle. These values could provide a relevant foundation for the development and implementation of new eHealth technologies. A total of 11 relevant patient values were identified, ranging from the need for security, support, and reduction in anxiety, to tailoring of treatment, and personalized and accessible care. We also showed a high consensus regarding the perceived importance of these values among patients with CVD.

The highest importance ratings were related to preserve a sense of autonomy, have or maintain a healthy lifestyle, and to have confidence and self-efficacy in the treatment and ability to achieve goals. According to a study by Zhang and colleagues [15] on health-related goals of patients with heart failure and self-care management, maintaining autonomy is the major patient goal [15]. This was evidenced by patient’s need to control their own lives and be physically independent [15]. The results of study 1 also showed that patients value security and support in reaching their health-related behavior goals: they need to know that they have the capacity (self-efficacy) to succeed or will be helped and guided. This feeling of security and perceived support has been highlighted in other studies on patients with CVD [16]. It is shown that patients with lower levels of self-efficacy in exercising are less physically active, regardless of their motivation to exercise [17]. Self-efficacy is also associated with better self-care [17] and adherence to healthy habits [15]. For patients with other chronic diseases, such as chronic obstructive pulmonary disease, values similar to ours have been identified, such as the value to receive empathy and be heard [18], to receive encouragement to be active [19] and to be reassured that exercising is safe [18,19]. In addition, it was reported in a study focusing on patients with heart failure that experiencing social support contributes to healthy self-care behavior [20], although the social environment can also affect the patients’ behavior in an unhealthy way [21]. Additionally, easy access to care (value 8) was highlighted as very important to support self-care of patients with CVD [22].

Of significance is the rating of value 9 (To be extrinsically motivated to accomplish goals or activities [related to health/lifestyle]), which differed from the ratings of the other values, with a rating distribution displaying less perceived importance than the distribution of the other values. Other studies show the complexity of motivation and rewards. Patients could be motivated by extrinsic motivations. However, other values could also indirectly act as a driving force to motivate patients to perform certain behaviors [23]. The Self-Determination Theory describes that needs such as competence, relatedness, and autonomy form the basis for self-motivation [24]. Thus, motivating patients using an extrinsic route may contribute to achieving a healthy lifestyle, but it may hamper the fulfillment of autonomy, which is a value that scored highly on importance in this study. Having a sense of autonomy can indirectly motivate patients to engage with the eHealth platform and self-manage their condition. The relation between values and outcomes is complex and fulfilling one value may not always mean that other values are reinforced too, even when target behaviors are the same.

### Recommendations

The patient values that were identified in this study can be used as input for the development or improvement of eHealth technologies aimed at patients with CVD. Even though the values were recognized and validated by a large patient group, the technical features that can be created based on these values may not be accepted by all patients. We recommend that the identified values are used as a starting point when setting requirements and designing technical features. When translating values into technology features and personalization of interventions, developers will still need to consider variation in preferences and personal circumstances. Overall goals and desires may be similar, but the exact approach will differ for each patient. Finding a workable level of personalization needs further study [25]. How values as a design basis can be integrated into technology is shown in a recent study by Asbjørnsen et al [26]. The authors combined different development and design approaches [8,27] with persuasive features [28] and behavior change techniques [29] to organize the development and design process of a weight loss maintenance interventions. The study shows how values can aid in operationalization of intervention components, to create focus and prioritize features and evaluate them based on stakeholder perspectives [26].

Additionally, patients’ values and behavior may change over time. Developers must consider that acceleration and dosage per value ingredient can both differ between patients and change over time for individuals. Therefore, one strategy is to let patients prioritize preset values or options to identify what appeals to them in treatment. One benefit of eHealth technology is that it can be tailored to patient’s wishes and needs and be sensitive to their underlying needs. Thus, patient data registered via the technology (eg, log data) may be used to further tailor the technology using predictive analyses or artificial intelligence. This way, certain behaviors or behavior changes can be predicted, and eHealth technologies could be adapted to them by providing the patient with information, support, or tasks [30].

In addition, when prioritizing the identified 11 values, patients were inclined to assess all values as important. Actually, some of these are quite basic human needs, and should be considered in any eHealth intervention. For example, catering to the value of staying healthy or being autonomous is interwoven with most eHealth designs. Thus, such important values should be considered in the design and researchers should aim to translate them into specific technology requirements [10]. Possibly, the more basic needs values leave more room for creative translations and more diverse requirements than more concrete specified values. There are many ways to operationalize an eHealth technology design that aims to keep users healthy, whereas the value of receiving information/being informed is already more specific. This is beyond the scope of this research, but we recommend evaluating eHealth interventions regarding how patient values are integrated into the technology. Future research should focus on whether and to what extent the integrated features/attributes within eHealth interventions contribute to patient (user) values, and determine which features are suitable for fulfilling specific patient values.

### Strengths and Limitations

A strength of the study is that by conducting usability tests, the study was not only able to identify patient perspectives, but also user perspectives (from patients using the BENEFIT PHP). Although usability tests have the purpose of recommending specific improvements for the design of eHealth interventions, this method is also useful to discover the drive of the users, their response to the technology, and the associated care tasks. Applying a think-aloud protocol during usability testing is useful for developing the design of the technology [31].

This study also had limitations. In the translation of needs and wishes into values, an interpretation bias may have occurred due to the qualitative nature of the data. To overcome this, a coding scheme was created based on a preliminary analysis and tested by 2 coders applying it and discussing disagreements. Another possible bias lies in the representativeness of the interview sample. The relatively low number of participants in study 1, with an overrepresentation of women may not generate results applicable to all patients with CVD. We have accounted for this bias and heterogeneity to some extent by validating the qualitative interview data among a large sample of patients with CVD. Even though they could not propose new values, the presented values were recognized by this group and except one—to be extrinsically motivated to accomplish goals or activities (related to health/lifestyle)—all values were perceived as important.

### Conclusion

When making design choices during the development of an eHealth technology, knowing how values are prioritized by patients may help in deciding if and how to implement features. Health care providers and patients can discuss which features match their needs and receive a more personalized approach from the technology. In addition, establishing a business model is very relevant for developers in making design choices. Next to considering which values contribute to the intended aim of the technology, another consideration is whether all proposed design choices based on values are feasible, affordable, and relevant for all key stakeholders.

## Appendix

Multimedia Appendix 1 Interview Script.

## Abbreviations

CVD: cardiovascular disease
PHP: personal health platform

## References

[1] F Piepoli Massimo (2017). 2016 European Guidelines on cardiovascular disease prevention in clinical practice : The Sixth Joint Task Force of the European Society of Cardiology and Other Societies on Cardiovascular Disease Prevention in Clinical Practice (constituted by representatives of 10 societies and by invited experts). Int J Behav Med.

[2] Cruz-Martínez Roberto Rafael, Wentzel J, Asbjørnsen Rikke Aune, Noort PD, van Niekerk JM, Sanderman R, van Gemert-Pijnen JEWC (2020). Supporting Self-Management of Cardiovascular Diseases Through Remote Monitoring Technologies: Metaethnography Review of Frameworks, Models, and Theories Used in Research and Development. J Med Internet Res.

[3] de Silva D (2011). Helping People Help Themselves: A Review of the Evidence Considering Whether It Is Worthwhile to Support Self-Management.

[4] Vervloet Marcia, Korevaar Joke C, Leemrijse Chantal J, Paget John, Zullig Leah L, van Dijk Liset (2020). Interventions to Improve Adherence to Cardiovascular Medication: What About Gender Differences? A Systematic Literature Review. Patient Prefer Adherence.

[5] Michie S, Yardley L, West R, Patrick K, Greaves F (2017). Developing and Evaluating Digital Interventions to Promote Behavior Change in Health and Health Care: Recommendations Resulting From an International Workshop. J Med Internet Res.

[6] Engelen MM, van Dulmen S, Puijk-Hekman S, Vermeulen H, Nijhuis-van der Sanden MW, Bredie SJ, van Gaal BG (2020). Evaluation of a Web-Based Self-Management Program for Patients With Cardiovascular Disease: Explorative Randomized Controlled Trial. J Med Internet Res.

[7] Van Velsen L, Wentzel J, Van Gemert-Pijnen JEWC (2013). Designing eHealth that Matters via a Multidisciplinary Requirements Development Approach. JMIR Res Protoc.

[8] Oinas-Kukkonen H, Harjumaa M (2009). Persuasive Systems Design: Key Issues, Process Model, and System Features. Communications of the Association for Information Systems.

[9] ten Klooster I, Wentzel J, Sieverink F, Linssen G, Wesselink R, van Gemert-Pijnen L (2021). Personas for Perfectly Tailored eHealth Technologies: A Multi-Method Study. JMIR Preprints.

[10] Keesman M, Janssen V, Kemps H, Hollander M, Reimer WSO, Gemert-Pijnen LV, Hoes A, Kraaij W, Chavannes N, Atsma D, Kraaijenhagen R, Evers A, BENEFIT consortium (2019). BENEFIT for all: An ecosystem to facilitate sustained healthy living and reduce the burden of cardiovascular disease. Eur J Prev Cardiol.

[11] Cruz-Martínez RR, Sieverink F, Wesselink R, van Gemert-Pijnen JEWC (2018). Towards Data-Driven Persuasive Coaching in a Heart Failure Telemonitoring Technology.

[12] Liederman Eric M, Morefield Catrina S (2003). Web messaging: a new tool for patient-physician communication. J Am Med Inform Assoc.

[13] IBM Corporation (2019). IBM SPSS Statistics for Windows, Version 26.0.

[14] Bolijn R, Schalkers I, Tan HL, Kunst AE, van Valkengoed IGM (2020). Patient perspectives on priorities for research on conventional and sex- and gender-related cardiovascular risk factors. Neth Heart J.

[15] Zhang KM, Dindoff K, Arnold JMO, Lane J, Swartzman LC (2015). What matters to patients with heart failure? The influence of non-health-related goals on patient adherence to self-care management. Patient Educ Couns.

[16] Klompstra L, Jaarsma T, Strömberg A (2018). Self-efficacy Mediates the Relationship Between Motivation and Physical Activity in Patients With Heart Failure. J Cardiovasc Nurs.

[17] Kessing Dionne, Denollet Johan, Widdershoven Jos, Kupper Nina (2016). Psychological Determinants of Heart Failure Self-Care: Systematic Review and Meta-Analysis. Psychosom Med.

[18] Papandony M, Chou L, Seneviwickrama M, Cicuttini F, Lasserre K, Teichtahl A, Wang Y, Briggs A, Wluka A (2017). Patients' perceived health service needs for osteoarthritis (OA) care: a scoping systematic review. Osteoarthritis Cartilage.

[19] Rodgers S, Dyas J, Molyneux A, Ward M, Revill S (2007). Evaluation of the information needs of patients with chronic obstructive pulmonary disease following pulmonary rehabilitation: a focus group study. Chron Respir Dis.

[20] Graven LJ, Grant JS, Vance DE, Pryor ER, Grubbs L, Karioth S (2015). Predicting Depressive Symptoms and Self-care in Patients with Heart Failure. Am J Hlth Behav.

[21] Buck HG, Mogle J, Riegel B, McMillan S, Bakitas M (2015). Exploring the Relationship of Patient and Informal Caregiver Characteristics with Heart Failure Self-Care Using the Actor-Partner Interdependence Model: Implications for Outpatient Palliative Care. J Palliat Med.

[22] Liljeroos M, Agren Susanna, Jaarsma T, Strömberg Anna (2014). Perceived caring needs in patient-partner dyads affected by heart failure: a qualitative study. J Clin Nurs.

[23] Jaarsma T, Cameron J, Riegel B, Stromberg A (2017). Factors Related to Self-Care in Heart Failure Patients According to the Middle-Range Theory of Self-Care of Chronic Illness: a Literature Update. Curr Heart Fail Rep.

[24] Deci EL, Ryan RM, Van Lange PAM, Kruglanski AW, Higgins ET (2012). Self-determination theory. Handbook of Theories of Social Psychology.

[25] van Velsen L, Broekhuis M, Jansen-Kosterink S, Op den Akker Harm (2019). Tailoring Persuasive Electronic Health Strategies for Older Adults on the Basis of Personal Motivation: Web-Based Survey Study. J Med Internet Res.

[26] Asbjørnsen RA, Wentzel J, Smedsrød ML, Hjelmesæth J, Clark MM, Solberg Nes L, Van Gemert-Pijnen JEWC (2020). Identifying Persuasive Design Principles and Behavior Change Techniques Supporting End User Values and Needs in eHealth Interventions for Long-Term Weight Loss Maintenance: Qualitative Study. J Med Internet Res.

[27] British Design Council (2005). The Double Diamond: A Universally Accepted Depiction of the Design Process.

[28] van Gemert-Pijnen JEWC, Nijland N, van Limburg M, Ossebaard HC, Kelders SM, Eysenbach G, Seydel ER (2011). A holistic framework to improve the uptake and impact of eHealth technologies. J Med Internet Res.

[29] Michie S, Johnston M (2012). Theories and techniques of behaviour change: Developing a cumulative science of behaviour change. Health Psychology Review.

[30] Michie S, Thomas J, Johnston M, Aonghusa PM, Shawe-Taylor J, Kelly MP, Deleris LA, Finnerty AN, Marques MM, Norris E, O'Mara-Eves Alison, West R (2017). The Human Behaviour-Change Project: harnessing the power of artificial intelligence and machine learning for evidence synthesis and interpretation. Implement Sci.

[31] Kilsdonk E, Peute L, Riezebos R, Kremer L, Jaspers M (2016). Uncovering healthcare practitioners' information processing using the think-aloud method: From paper-based guideline to clinical decision support system. Int J Med Inform.

