# Supplementary material for: Values of Importance to Patients With Cardiovascular Disease as a Foundation for eHealth Design and Evaluation: Mixed Methods Study
Source: JMIR Cardio. 2021 Oct 22;5(2):e33252. doi: 10.2196/33252 (PMC8571692; doi:10.2196/33252)
Supplement: Multimedia Appendix 1 [file cardio_v5i2e33252_app1.docx]

# Appendix 1: Interview Script

The interviews were conducted between June and August of 2018. The average duration of each interview was around 45 minutes to 1 hour.

The script used for the interviews consisted of 4 parts, an introduction, the interview, the usability test, and some final questions.

The table below shows the actions, goals, and further elaboration on the goals.

| **Actions** | **Goal(s)** | **Elaboration:** |
| --- | --- | --- |
| Introduction | -Getting acquainted with each other, easing the mood of the interviewer and the participant.  -Introduction to the interview.  -Explaining the goal of the interview.  -Singing the informed consent.  -Checking that the interview is properly recorded. | -Using small talk.  -No usage of jargon.  -Making sure the participant cannot give any wrong answers. |
| Interview | -Getting insight in personal and medical background  -Getting insight in rehabilitation process  -Getting insight in the adjustments in lifestyle due to the illness  -Getting insight in self-care  -Getting insight in communication with health care providers  -Getting insight in personal motivation for rehabilitation | -The interview consisted of around 13 questions, but since the interviews were deliberately of an open nature, the emphasis of the conversation could shift depending on the situation. |
| Usability test | -Getting the participant to perform certain tasks on the case example platform.  -Examining situations where things were unclear, or in which the participant got stuck.  -Finding out what the participants thought of the platform and how they thought it could aid their rehabilitation. | -Very broad assignments were given without further instructions, to see how the participants would navigate through the platform.  -During the entire process the participants were being asked why they would perform certain actions.  -Questions about how eHealth in general could benefit rehabilitation were also asked. |
| Final questions | -Getting the participants opinion of the specific case example platform.  -Getting the participants opinion on how eHealth can fit in the lives of the participants.  -Closing the interview and thanking the participants for their time. | -What questions still need to be asked is dependent on the information the participants have already given during the interview / usability test. |
